# The effect of light supplementation on the changes in outer and inner fruit quality of different *Selenicereus* spp. cultivars

**DOI:** 10.1002/jsfa.70914

**Published:** 2026-07-29

**Authors:** Luíz Guilherme Malaquias da Silva, Carlos Alexandre Rocha da Costa, Giovanni Aleixo Batista, Estela Corrêa de Azevedo, Fabrício Teixeira de Lima Gomes, Laryssa Ribeiro Calcagnoto, Gabriel Luis Castiglioni, Marcelo Ângelo Cirillo, Danilo José Machado de Abreu, Flávia Matias Oliveira da Silva, Katiúcia Alves Amorim, Leila Aparecida Salles Pio, Eduardo Valério de Barros Vilas Boas, Elisângela Elena Nunes Carvalho

**Affiliations:** ^1^ Department of Food Science Federal University of Lavras (UFLA) Lavras Brazil; ^2^ Department of Soil Science Federal University of Lavras (UFLA) Lavras Brazil; ^3^ Department of Statistics Federal University of Lavras (UFLA) Lavras Brazil; ^4^ School of Agronomy of the Federal University of Goiás Goiânia Brazil; ^5^ Biology Department Federal University of Lavras Lavras Brazil; ^6^ Polytechnic Institute of Beja, Higher School of Agriculture, Rua Pedro Soares Beja Portugal; ^7^ MED – Mediterranean Institute of Agriculture, Environment and Development CHANGE – Institute for Global Change and Sustainability, University of Évora, Polo da Mitra Évora Portugal; ^8^ Department of Agriculture Federal University of Lavras Lavras Brazil

**Keywords:** antioxidants, artificial lighting, fruit quality, LEDs, primary and secondary metabolites

## Abstract

**BACKGROUND:**

*Selenicereus* spp., belonging to the Cactaceae family, produces pitaya, a fruit recognized for its exotic appearance and rich phytochemical composition. In regions with shorter photoperiods, flowering and fruit development are limited, making light supplementation a promising strategy. However, its effects on fruit quality, particularly across different cultivars, remain insufficiently explored. This study aimed to evaluate the influence of LED light supplementation during flowering on the physicochemical characteristics and bioactive compounds of pitaya fruits.

**RESULTS:**

Plants of ‘Boreal Red’ and ‘Chinesa’ (*S. undatus*) and ‘Roxa do Pará’ (*S. costaricensis*) were subjected to light supplementation for 4 h daily (20:00–24:00) during the flowering period (20 ± 3 days) using full‐spectrum LED lamps (400–700 nm). Fruits were harvested at commercial maturity and analyzed for physical, physicochemical, and biochemical attributes. Light supplementation significantly increased fruit mass (19.04%), peel brightness (4.19%), peel chromaticity (5.31%), pulp hue (66.65%), fructose (29.17%), and vitamin C (6.73%) across cultivars. Additionally, ‘Boreal Red’ exhibited increases in pulp hue (30.67%), sucrose (23.19%), and glucose (30.12%).

**CONCLUSION:**

Light supplementation during flowering positively influences fruit quality attributes in pitaya, enhancing both primary metabolites such as sugars, and bioactive compounds such as vitamin C. These findings support the use of light supplementation as a strategy to improve fruit quality under short photoperiod conditions, contributing to the development of more efficient cultivation practices in Brazil. © 2026 The Author(s). *Journal of the Science of Food and Agriculture* published by John Wiley & Sons Ltd on behalf of Society of Chemical Industry.

## INTRODUCTION

Belonging to the Cactaceae family and produced by species of the genus *Selenicereus*, pitaya is an exotic tropical fruit that stands out for its vibrant appearance and nutritional properties.^1^ Rich in potassium, magnesium, calcium, iron, and copper, pitaya also contains essential amino acids such as histidine, lysine, methionine and phenylalanine, and B vitamins, proteins, and fibers.^2^, ^3^ The flowering of the plant is favored by long days and floral anthesis occurs at night, lasting between 9 and 14 h, ending at dawn.^4^ After flowering and pollination – an essential phase of reproductive development – the flowers wither.^5^, ^6^

Edaphoclimatic and seasonal conditions in Brazil directly influence pitaya production, particularly through reductions in photoperiod during certain times of the year. Under these short‐day conditions, flowering is suppressed, resulting in harvest and off‐season periods.^7^ Temperature also plays an important role, with optimal growth occurring between 25 and 35 °C, while temperatures below 10 °C or above 38 °C interrupt plant development.^8^ In major producing states such as São Paulo, Santa Catarina, Minas Gerais, and Paraná, fruit production is generally interrupted from April to August, when day length ranges from 11 to 11.9 h.^9^ However, flowering can be induced when plants enter the reproductive phase under appropriate light conditions.^10^

In this context, nighttime light supplementation (LS) becomes a promising alternative at the beginning of the reproductive phase, which can be stimulated and intensified by LS using photosynthetically active radiation.^7^, ^11^, ^12^ Wang *et al*.^8^ reported that LEDs applied during winter nights can interact with endogenous hormonal signaling in pitaya, particularly cytokinin, which regulates bud formation, and gibberellic acid, which promotes bud development. Therefore, this technology influences the production of plant hormones essential for flowering induction and fruit development.^13^, ^14^

Studies using *Slenicereus* as a model cultivar to investigate artificial lighting for flowering induction and off‐season production have demonstrated that this technology is promising and applicable.^15^, ^16^, ^17^ Previous studies also reported increases in pulp yield and soluble solids content in several pitaya cultivars and clones from Central America, Taiwan, and Vietnam, including ‘Orejona’, ‘Lisa’, ‘Rosa’, ‘Jhubei 3’, ‘D4’, ‘D15’, ‘D18’, and ‘Small Nick’,^18^ as well as improvements in the quality of the cultivar ‘Zimilong’.^19^ Therefore, LS under short photoperiod conditions represents a promising strategy to improve fruit development and quality, contributing to the economic value of pitaya production.

Although studies have reported the effects of LS in different species, there is no scientific evidence regarding its effects on *Selenicereus* spp. cultivated in Brazil. Therefore, this study aimed to evaluate the influence of LS during flowering on the fruit quality of three *Selenicereus* spp. cultivars.

## MATERIALS AND METHODS

### Experimental design and plant material

Plants of the cultivars ‘Boreal Red’ (A), ‘Chinesa’ (B) (*S. undatus*) and ‘Roxa do Pará’ (C) (*S. costaricensis*) (Fig. 1) were subjected for 4 h (from 20:00 to 00:00) daily during the flowering period (20 days) to LS (Fig. 2), using lamps composed of light‐emitting diodes/full‐spectrum photons, between 400 and 700 nm (LED lamps), capable of activating photosynthetically active radiation measured in μmol m^−2^ s^−1^. Artificial management of the photoperiod was carried out on 1 ha at the Nascente da Pitaya & Cia site, located in Bambuí, MG, Brazil (latitude: 20° 00′ 21″ S; longitude: 45° 58′ 37″ W). All the energy from the LS consumed during flowering came from the property's solar panels. To evaluate the results, control data were collected from cultivars grown outside the LS system, maintaining the same local conditions.

**Figure 1:**
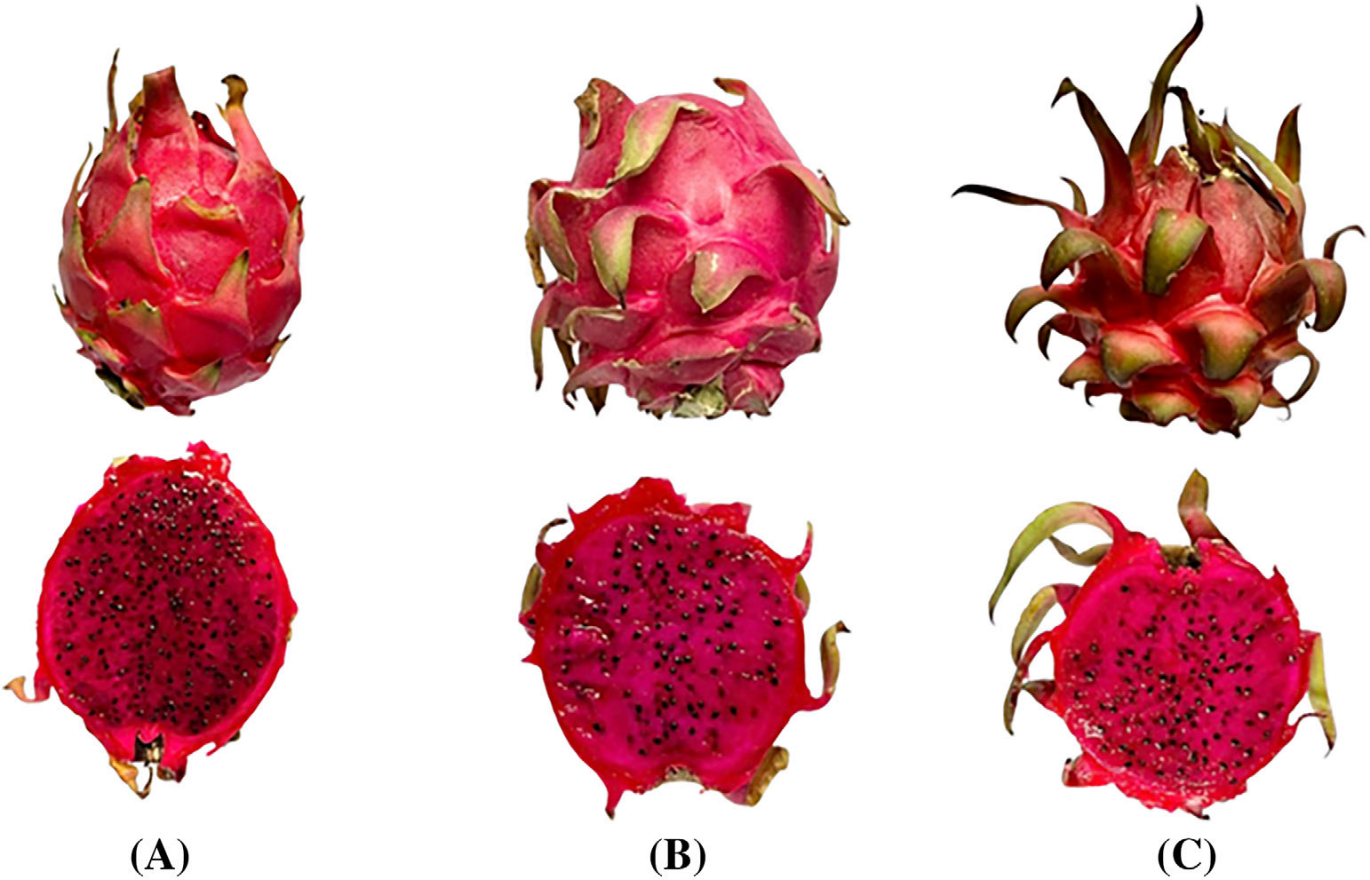
Pitaya fruit cultivars: (A) ‘Boreal Red’; (B) ‘Chinesa’ (*Selenicereus undatus*); (C) ‘Roxa do Pará’ (*Selenicereus costaricensis*).

**Figure 2:**
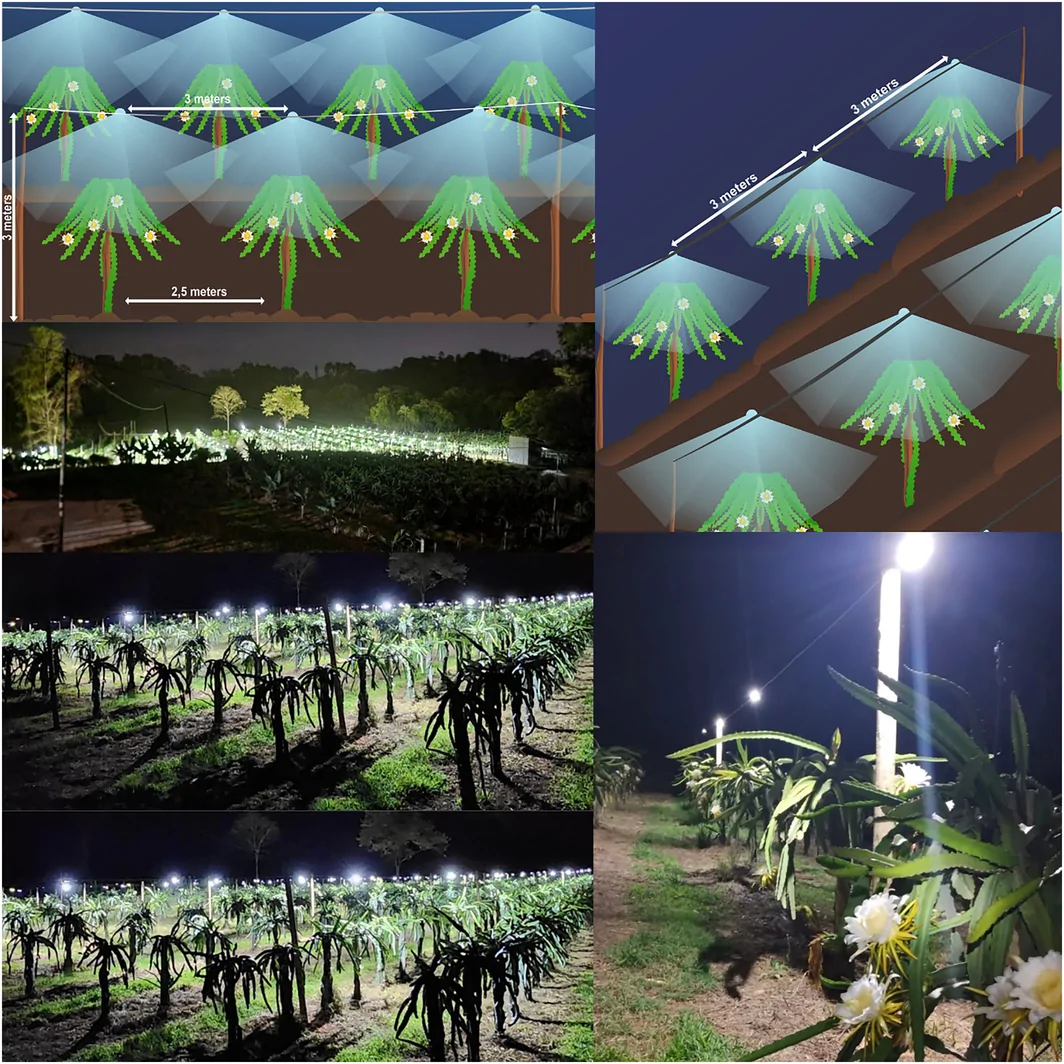
Diagram of the experimental field with the implementation of light supplementation.

Fruits were harvested at commercial maturity, when the scales were half green and half pink. Fruits with visible defects or signs of pathogen infection were discarded, and the remaining samples were transported in insulated expanded polystyrene boxes with dry ice to the Fruit and Vegetable Postharvest Pilot Plant at the Federal University of Lavras, Lavras, MG, Brazil. The fruits were washed, sanitized in 1% sodium hypochlorite for 2 min, rinsed with distilled water, and dried at room temperature. Each experimental unit consisted of 10 fruits, representing four independent biological replicates per treatment. Composite samples were prepared by homogenizing the edible portion of the fruits from each replicate, and all analyses were performed in quadruplicate. The peel (mesocarp and exocarp) and pulp (endocarp)were separated, packaged in low‐density polyethylene bags, and stored at −80 °C until analysis.

### Physicochemical analysis

The pH of the pulp was measured using a pH meter (R‐TEC‐7‐MP, Tecnal, Piracicaba, SP, Brazil). The titratable acidity of the fruits was determined according to the AOAC methods,^20^ by titration with sodium hydroxide (NaOH) 0.1 mol L^−1^ using phenolphthalein as an indicator, and the results were expressed as the percentage of malic acid in fresh mass. The soluble solids contents of the pulp without dilution were obtained using a digital refractometer (PR‐100, Atago, Tokyo, Japan), and the results were expressed as a percentage.^20^ The mass was determined by weighing the fruits using an analytical balance (Marte D330, Marte Científica, São Paulo, Brazil), and the results were expressed in grams. The pulp yield was calculated as the ratio of the mass of the whole fruits to the mass of the pulp, with the results expressed as a percentage. For the peel and pulp color of each cultivar, the evaluation was performed in its equatorial region using a colorimeter (CR‐400, Konica Minolta, Tokyo, Japan) in the color space of the International Commission on Illumination, employing the L*, a*, and b* color scale system, chroma (C*) and hue angle (*h*°). Firmness was determined using a texture analyzer (TA.XT2i, Stable Micro Systems Ltd, Godalming, UK), using a cylindrical probe (2 mm in diameter) with a test speed equal to 1 mm s^−1^, at a penetration distance of 3 mm, with the HDP/90 platform used as a base. The results for peel and pulp were expressed in newtons (N).

### Fruit dimensions

The length, diameter and thickness of the exocarp, and area of the fruits were obtained digitally with the aid of an ImageJ processor, measured directly on the images using a scale of 1 cm.^21^ The results were expressed in centimeters.

### Fructose, glucose, and sucrose content

The sugars fructose, glucose and sucrose were quantified by high‐performance liquid chromatography (HPLC) according to the methodology described by Santos *et al*.^22^ Approximately 1 g of pitaya pulp was diluted in 5 mL of ultrapure water. After homogenization of the samples, 1 mL was collected for 2× dilution with ultrapure water and analyzed in a chromatograph (Prominence model, Shimadzu, Kyoto, Japan), with a Shim‐pack SCR 102HG column protected by an SCR 102HG pre‐column. The substances were visualized using refractive index (RID‐20 A) and UV–visible (SPD‐20A) detectors, maintaining the oven temperature at 50 °C and a mobile phase flow of 5 mmol L^−1^ perchloric acid at 0.600 mL min^−1^. The results were expressed as g kg^−1^ of fresh weight (FW).

### Bioactive compounds and antioxidant activity

#### Extraction for bioactive compounds

To obtain extracts for the determination of bioactive compounds, 2 g of the sample was homogenized with 20 mL of 50% methanol (v/v), subjected to an ultrasonic bath (Dubnoff orbital bath, model NT 230, Novatecnica, Piracicaba, SP, Brazil) for 30 min at room temperature, and centrifuged (Rotina 380R, Andreas Hettich, Tuttlingen, Germany) for 15 min. The supernatant was filtered using quantitative filter paper (12.5 cm diameter, 0.025 mm porosity). A second extraction was performed with 20 mL of 50% acetone (v/v) under the same conditions, and the extracts were combined to maximize the recovery of bioactive compounds, as previously demonstrated for pitaya matrices using solvent systems with different polarities.^23^

#### Betalain content

The betalain (betacyanin and betaxanthin) content was determined according to a method described by Qingzhu *et al*.,^12^ modified by Zitha *et al*.^24^ Readings were performed using a microplate reader (EZ Read 2000, Biochrom, Cambridge, UK) and the results were expressed as mg kg^−1^ FW.:

$$\text{Betacyanin}\;\left({\mathrm{mg}}^{-1}\right)=\frac{A_{538}\times \mathrm{DF}\times \mathrm{MM}\times V\times 100}{\varepsilon \times P\times l}$$

$$\text{Betaxanthin}\;\left({\mathrm{mg}}^{-1}\right)=\frac{A_{483}\times \mathrm{DF}\times \mathrm{MM}\times V\times 100}{\varepsilon \times P\times l}$$

where *A*_538_ is the absorbance for betacyanin at 538 nm; *A*_483_ is the absorbance for betaxanthins; DF is the dilution factor; MM is the molecular mass (550 g mol^−1^ for betanin and 308 g mol^−1^ for indicaxanthin); *V* is the volume of the pigment solution (mL); *Ɛ* is the molar extinction coefficient (60 000 L mol cm^−1^ for betanin and 48 000 L mol cm^−1^ for indicaxanthin); *l* is the path length (1 cm); and *P* is the mass of fresh pigment (mg). All pigments were measured using a microplate reader (Biochrom EZ Read 2000).

#### Total flavonoids and total phenolic compounds

The total flavonoid content (TFC) was determined through spectrophotometric assay with aluminum chloride, with adaptations.^25^ A 125 μL sample of the extract was added to a test tube (10 mL) together with 75 μL of sodium nitrite (5% m/v). After 6 min of reaction at rest in the dark, 150 μL of aluminum chloride (10% m/v) was added, and the mixture remained at rest for 5 min. Subsequently, 750 μL of sodium hydroxide (1 mol L^−1^) and 1400 μL of distilled water were added. After homogenization using a vortex (K45‐2810, Kasvi, Pinhais, PR, Brazil) and incubation for 15 min, absorbance was measured at 514 nm using a microplate reader (Biochrom EZ Read 2000). The results were expressed as milligrams of quercetin equivalents per kilogram of fresh weight (mg QE kg^−1^ FW).

Total phenolic content (TPC) was determined by the Folin–Ciocalteu method,^26^ with adaptations by da Silva *et al*.^27^ A mixture of 3 μL of the extract +150 μL of Folin–Ciocalteu reagent (10% v/v) + 120 μL of Na₂CO₃ (4% m/v) was allowed to react for 2 h at room temperature (25 °C) in the dark. Quantification was performed spectrophotometrically (Biochrom EZ Read 2000) at 720 nm. The results were expressed as milligrams of gallic acid equivalents per kilogram of fresh weight (GAE kg^−1^ FW).

#### Antioxidant activity

Antioxidant activity was determined by the 2,2′‐azino‐bis(3‐ethylbenzothiazoline‐6‐sulfonic acid) (ABTS) assay according to Rufino *et al*.,^28^ Kolap *et al*.,^29^ and Monteiro *et al*.^30^ The ABTS^+^ solution was diluted with ethanol to an absorbance of 0.700 ± 0.02 at 734 nm. For the determination, 30 μL of sample was added to 3 mL of diluted ABTS^+^ solution, and absorbances were recorded 6 min after mixing. Ascorbic acid solutions (1–6 mg mL^−1^) were used as standard and positive controls and the results were expressed as percent reduction:

$${\text{ABTS}}^{+}\;\text{reduction}\left(\%\right)=\frac{\text{Absorbance of control}-\text{Abssorbance of sample}\times 100}{\text{Absorbance of control}}$$

Determination of the phosphomolybdenum complex (PC) was performed according to the methodology described by Prieto *et al*.,^31^ with adaptations. A volume of 0.5 mL of the samples was pipetted in triplicate into screw‐cap tubes. Subsequently, 1.5 mL of the phosphomolybdenum reagent was added, and the tubes were gently mixed for 10 s. The tubes were then incubated in a water bath for 90 min. After cooling in an ice bath, absorbance was measured using a spectrophotometer (Biochrom EZ Read 2000) at 695 nm. The results were expressed as milligrams of ascorbic acid equivalents per kilogram of fresh weight (mg AA kg^−1^ FW).

#### Vitamin C

Vitamin C was quantified according to the method proposed by Strohecker *et al*.^32^ Absorbance was measured at 520 nm using a 96‐well microplate reader (Biochrom EZ Read 2000). The results were expressed as milligrams of ascorbic acid per kilogram of fresh weight (mg AA kg^−1^ FW).

### Statistical analysis

The experiment was conducted in a completely randomized design in a 2 × 3 factorial arrangement, with lighting – absence of light supplementation (ALS) and presence of lighting (LS) – and cultivar (‘Boreal Red’, ‘Chinesa’, and ‘Roxa do Pará’) as factors. Each treatment consisted of four replicates, totaling 24 experimental units.

Data were subjected to ANOVA to evaluate the effects of LS, cultivar, and their interaction. When significant effects were detected, means of the lighting factor were compared by the *F*‐test, whereas means of cultivars were compared using Tukey's test at a significance level of 5% (*P* < 0.05).

To investigate the relationships among the physicochemical and biochemical variables and the effects of cultivar and LS, multiple factor analysis for contingency tables (MFACT) was performed^33^, ^34^, ^35^. The results were visualized using perceptual maps and correlation circles. All analyses were conducted in the RStudio environment.

## RESULTS AND DISCUSSION

Table 1 presents the average results of the characterization of pitayas (‘Boreal Red’, ‘Chinesa’, and ‘Roxa do Pará’) ALS and LS and, in Fig. 3, the results of principal component analysis (PCA), matrix of inertia/scores of the variables and correlation circle.

**Table 1:** General characteristics of pitaya fruits under the absence and presence of light supplementation (ALS and LS, respectively), considering all evaluated ***Selenicereus*** cultivars.

Variable	M (ALS)	SD (ALS)	CV (ALS)	M (LS)	SD (LS)	CV(LS)
pH	3.28	0.22	6.72	3.19	0.24	7.62
Acidity	0.28	0.01	4.93	0.28	0.02	6.52
Soluble solids	15.86	1.09	6.89	16.22	0.81	4.96
Vitamin C	355.9	10.85	30.49	379.9	10.80	28.42
Sucrose	14.2	0.16	11.12	14.7	0.20	13.84
Glucose	25.8	0.47	18.18	28.5	0.56	19.46
Fructose	36.0	0.38	10.55	46.5	1.13	24.37
TFC	262.0	5.41	20.65	274.6	5.77	21.00
TPC	2125.1	11.22	5.28	2172.3	7.80	3.59
PC	7945.0	209.14	26.32	7630.5	193.06	25.30
L. peel	43.26	1.56	3.60	45.06	1.58	3.52
C. peel	44.26	2.19	4.95	46.62	1.99	4.27
H. peel	17.04	3.55	20.85	23.97	4.37	18.25
L. pulp	23.73	3.39	14.28	25.59	1.82	7.09
C. pulp	23.24	8.18	35.19	30.75	4.17	13.56
H. pulp	6.31	1.76	27.84	10.59	1.77	16.69
Length	13.24	0.95	7.21	13.76	0.73	5.33
Width	7.70	0.46	5.97	8.57	0.83	9.67
Mass	399.16	77.99	19.54	495.79	92.42	18.64
Yield	68.48	5.47	7.99	64.79	2.52	3.89
Peel firmness	49.46	9.22	18.64	49.14	9.64	19.61
Pulp firmness	3.30	0.25	7.71	3.33	0.22	6.72
BTS	122.89	5.82	4.73	129.28	2.85	2.21
BTX	77.69	3.48	4.48	77.59	3.27	4.21
ABTS	43.29	3.95	9.11	47.04	6.28	13.36

M, mean; SD, standard deviation; CV, coefficient of variation; L, lightness; C, chroma; H, hue angle; BTS, betacyanins; BTX, betaxanthins; ABTS, 2,2′‐azino‐bis(3‐ethylbenzothiazoline‐6‐sulfonic acid).

**Figure 3:**
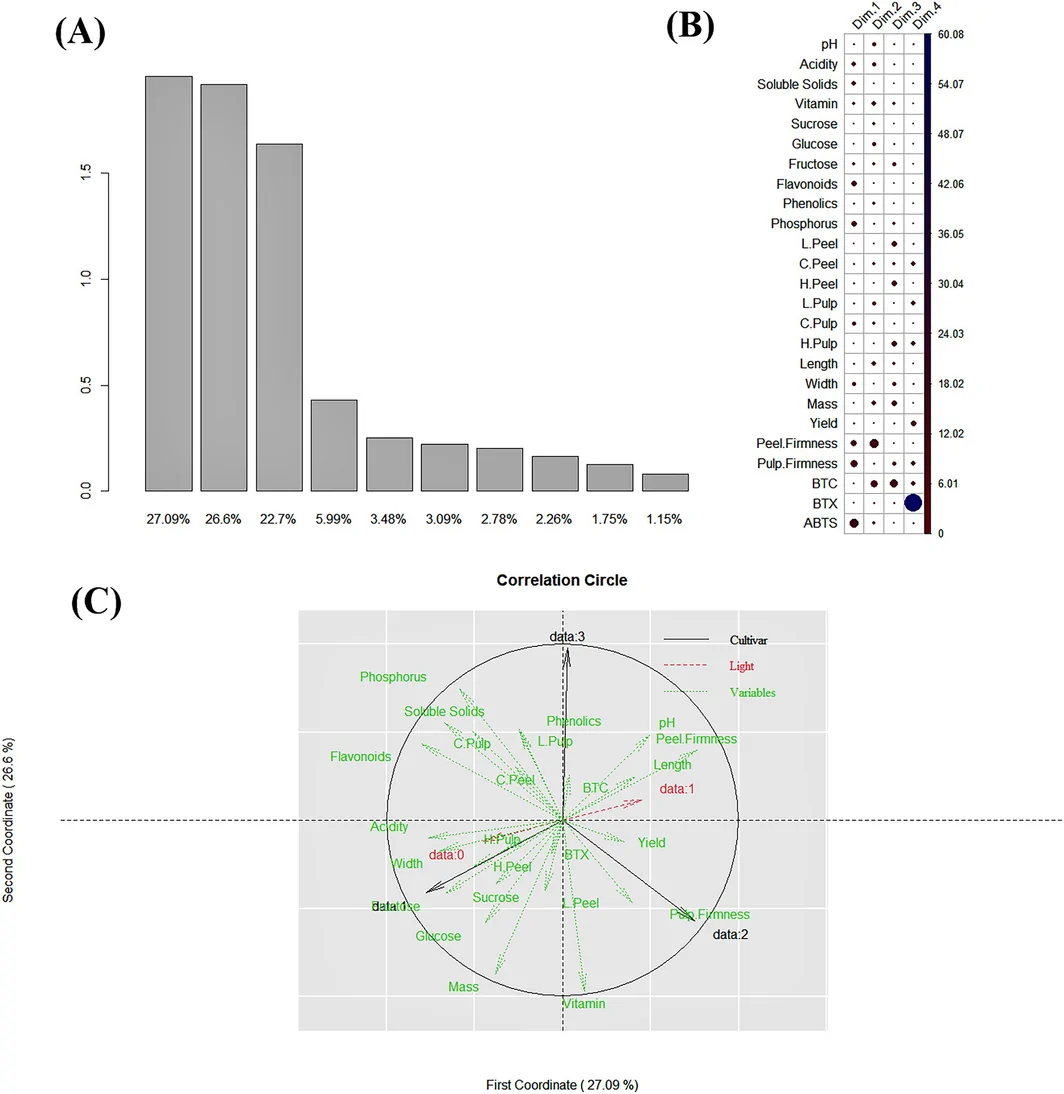
Results of principal component analysis (A), matrix of inertia/variable scores (B), and the correlation circle (C). Dim.1‐Dim.4 represent the principal component dimensions; L, lightness; C, chroma, H, hue angle; BTS, betacyanins; BTX, betaxanthins; ABTS, 2,2′‐azino‐bis(3‐ethylbenzothiazoline‐6‐sulfonic acid).

The coefficients of variation of the characteristics of pitaya fruit indicate significant variability among the samples. Vitamin C and glucose presented the highest coefficients, evidencing great variation in the concentrations of these components. TPC, betacyanidin and acidity recorded the lowest coefficients, which demonstrates greater uniformity among the samples.

The PCA results showed that the first four components explained 82.38% of the total variance, with the first two components accounting for 53.69%. The matrix of partial inertias indicated that the evaluated variables showed the highest contribution to the first component (0.9741), followed by cultivar (0.7770), whereas cultivar (0.9695) and the evaluated variables (0.9346) were the main contributors to the second component. LS showed a lower contribution to the first two components but became more relevant in the third component (0.7612), indicating its influence on additional sources of variation in fruit quality.

The correlation circle indicated that LS was associated with increases in fruit mass, peel and pulp color parameters, sugars (sucrose, glucose, and fructose), and vitamin C, whereas fruits grown without supplementation were more closely associated with betalains and pH, particularly in the cultivar ‘Boreal Red’.

Regarding LS, no statistically significant changes were observed in the titratable acidity of the cultivars under study. Regarding mass, there was an increase in all LS cultivars, although there was a statistically significant reduction in pulp yield in all cultivars analyzed (*P* < 0.05). These findings suggest that, although LS leads to an increase in fruit mass, it does not enhance pulp yield. This suggests that the increase in fruit mass may be associated with changes in peel thickness or pericarp structure, rather than a direct increase in pulp yield, although these parameters were not directly measured in the present study. Further analysis of the peel‐to‐pulp ratio and anatomical modifications could help clarify the effects of LS on fruit development. The yields obtained were lower than those reported by Magalhães *et al*.,^36^ who observed pulp yields greater than 75% after 36 days after anthesis, without LS for red pitaya fruit (*Selenicereus polyrhizus*).

LS increased the lightness of the pulp of ‘Boreal Red’ pitaya (*P* < 0.05). Regarding C*, the LS condition was effective in increasing the variable for the cultivars ‘Boreal Red’ and ‘Chinesa’, while ‘Roxa do Pará’ did not show significant differences. Regarding hue, LS significantly increased (*P* < 0.05) the *h*° value of the peels of the cultivars ‘Boreal Red’ and ‘Chinesa’, while it reduced the *h*° value of the cultivar ‘Roxa do Pará’.

The absence of significant differences in the dimensions of the cultivars analyzed may be related to a phenotypic similarity between *S. undatus* and *S. costaricensis*. This similarity may be the result of a sharing of morphological characteristics or that the cultivation environment exerted influences during the development of the fruits.^23^, ^37^

In a smaller fraction among the sugars determined, sucrose content in the cultivar ‘Boreal Red’ increased from 13.8 to 17.0 g kg^−1^ FW. In the ‘Chinesa’ cultivar, a decrease was observed from 15.6 to 12.7 g kg^−1^ FW, while ‘Roxa do Pará’ did not show significant differences (*P* < 0.05). Rocha *et al*.^38^ did not find sucrose values in the determination of sugars for *S. undatus* with white and red pulp. These results may be linked to different factors, such as the intensity and type of artificial light on each of the cultivars.^4^ According to Berk,^39^ sugars constitute approximately 80% of soluble solids, in which glucose and fructose are the main components of most fruits, while sucrose is more prevalent in certain species. Although artificial lighting is not applied directly to the fruit, it is possible that stress occurs in the plant under such conditions, affecting sugar production in the fruit. In agreement with our findings, Sun *et al*.^40^ observed higher total and reducing sugar contents in pitaya fruits exposed to supplemental blue light. These responses may be associated with changes in central metabolism, as supplemental light has been shown to regulate amino acid pathways and increase metabolic intermediates linked to fruit growth and nutrient accumulation.^40^ However, further studies to investigate the relationship between exposure time and light intensity and its relationship with sugar production should be investigated.

Sugars are essential metabolites contributing to fruit quality, as consumers generally prefer sweeter pitayas.^41^, ^42^ The results indicate that the glucose content of the ‘Boreal Red’ cultivar was positively affected by LS, reaching 35.0 g kg^−1^ FW, while the control showed 20.6 g kg^−1^ FW. The cultivars ‘Chinesa’ and ‘Roxa do Pará’ did not show statistically significant differences (*P* < 0.05) under the influence of LS. The differences in sugar levels between some cultivars and the increase in glucose under the influence of LS in the cultivar ‘Boreal Red’ may also be related to the genetic variability between the cultivars analyzed, as well as to the agricultural practices adopted.^37^

This study highlights that there were no significant differences (*P* < 0.05) for betacyanin and betaxanthin levels in all cultivars. These pigments are part of the large group of betalains, which give pitaya fruit its characteristic color.^43^ Although betaxanthin is the pigment with the lowest proportion in the red pulp of some cultivars, it adds to the other water‐soluble betamic pigments to make up the total betalain content.^44^, ^45^

Next, it was observed that LS as an induction during flowering demonstrated statistically significant effectiveness (*P* < 0.05) in relation to conventional cultivation. This can be considered when evaluating different variables that are correlated with fruit quality, such as mass, clarity and intensity of peel color and pulp hue, in addition to glucose, fructose, and vitamin C levels, for all cultivars.

The data obtained demonstrate that LS resulted in a statistically significant increase in fruit mass, with the cultivar ‘Boreal Red’ presenting the greatest increase at 38.10%, followed by the cultivars ‘Roxa do Pará’ and ‘Chinesa’, with increases of 20.30% and 9.50%, respectively. The observed results are in agreement with those of Tran *et al*.,^18^ who compared conventional fruits obtained from natural production in the summer of 2013 with those treated with LS, during the 2013–2014 crop season, in which an increase in the mass of the cultivars ‘Orejona’, ‘Rosa’, ‘Jhunbei 3’, ‘Chaozhou large’, ‘Chaozhou 5’ and ‘Small Nick’ was observed from 196.6 to 331.4 g, from 251.7 to 472.0 g, from 176.8 to 277.2 g, from 421.2 to 541.8 g, from 314.1 to 329.0 g, and from 288.2 to 350.5 g, respectively. Chen *et al*.,^19^ when evaluating the effect of blue–red light emitted by a diode on the differentiation of flower buds and the quality of pitaya fruit, observed that supplementation promoted the best performance in terms of number of buds and productivity, with an impressive average yield of 2.236 kg per plant. Similarly, Sun *et al*.^40^ reported that supplemental blue light increased pitaya fruit weight mainly through greater pulp accumulation during fruit development. According to da Silva *et al*.,^1^ the increase in fruit mass is associated with the development and maturation process, which includes the increase in water content, the accumulation of sugars and the maturation of seeds. This finding is extremely important for producers, since the stability of the mass can positively impact the standardization of the final product, essential for the consumer market.

The analysis of color parameters is essential to determine the quality and acceptance of fruits by consumers. Fruit color is influenced by several factors, including genetics, growing conditions, and degree of ripeness. According to the results obtained, it was observed that LS affects the brightness and intensity parameters in the peel and in the hue of the pulp of all cultivars under study. Research by Ntagkas *et al*.^46^ showed that different qualities of LED light resulted in variations in the amount of pigments in fruits, directly affecting color parameters.

The mean L* values for the peel increased from 43.25 to 45.06, under the influence of LS (*P* < 0.05). This fact may be correlated with variations in pigment synthesis or changes in the structure of the peel surface. In addition, there was also an increase in the hue of the fruit pulp from 6.31 to 10.58.

LS resulted in a statistically significant increase in the chromaticity of the peel of all pitaya fruit varieties analyzed, indicating that exposure to light can intensify the color of the peel. This effect may be related to changes in the concentration of pigments such as betalains present in pitaya fruit peels.^47^ LS may also play a key role in modulating the synthesis of secondary metabolites in pitaya fruits. Under natural short‐day conditions, reduced light exposure can limit carbon assimilation and the activation of metabolic pathways associated with the biosynthesis of phenolic compounds, flavonoids, betalains, and vitamin C. In contrast, artificial lighting, especially LED‐based systems, extends the effective photoperiod and can stimulate these biosynthetic pathways, resulting in enhanced accumulation of bioactive compounds. Previous studies have reported increases of up to 25% in total phenolics and 30% in carotenoids under LS, reinforcing its role in improving nutritional and functional attributes.^4^ This effect is often associated with the activation of light‐responsive regulatory mechanisms and enzymatic activity linked to plant photoreceptors. Therefore, the differences observed between fruits produced under LS and conventional conditions may reflect not only variations in primary metabolism, such as sugar accumulation, but also the modulation of secondary metabolism, which directly influences nutritional quality and antioxidant potential. Sun *et al*.^40^ also reported that supplemental blue light enhanced the accumulation of flavonoids and other bioactive compounds in pitaya fruits. According to Zhang *et al*.,^48^ the greatest accumulations of pigments responsible for color are observed in plants that receive greater incidence of light. For the fruit pulps, a statistically significant increase in chromaticity was observed for the ‘Boreal Red’ and ‘Chinesa’ varieties, while the ‘Roxa do Pará’ variety showed a reduction in the C* value (*P* < 0.05).

LS significantly increased the *h*° values of the pulp in all cultivars, indicating changes in color associated with the biosynthesis and accumulation of betalains in the peel and pulp. In addition to their role in fruit coloration, betalains are bioactive pigments with recognized antioxidant and health‐promoting properties.^19^, ^49^, ^50^, ^51^ Recent studies have shown that supplemental light can also enhance the accumulation of other bioactive compounds, including tocopherols and phenolic acids, reinforcing the broad effect of light on secondary metabolism.^40^

Regarding fructose, all cultivars under LS showed higher levels compared to ALS conditions. The cultivar ‘Boreal Red’ under LS reached 55.9 g kg^−1^ FW, while the control showed 40.3 g kg^−1^ FW. The cultivar ‘Chinesa’ presented 43.7 g kg^−1^ FW under LS and 33.9 g kg^−1^ FW under ALS, whereas ‘Roxa do Pará’ showed 38.1 g kg^−1^ FW under LS and 33.7 g kg^−1^ FW in the control. These values are similar to those reported for red‐fleshed pitayas (*S. undatus*) cultivated under conventional conditions (42.5 g kg^−1^ FW) and higher than those observed for *S. costaricensis* (27.7 g kg^−1^ FW).^38^, ^52^ The highlighted levels contribute to the sweet flavor of pitaya, making the fruit even more pleasant and with a palatability that is more appreciated, encouraging the consumption of nutritious foods that generate energy for cells.^41^, ^42^, ^53^

Analysis of these data was also performed using a two‐dimensional graph (Fig. 4), which illustrates the distribution of individuals and variables according to the main dimensions and highlights the effects of cultivar and LS.

**Figure 4:**
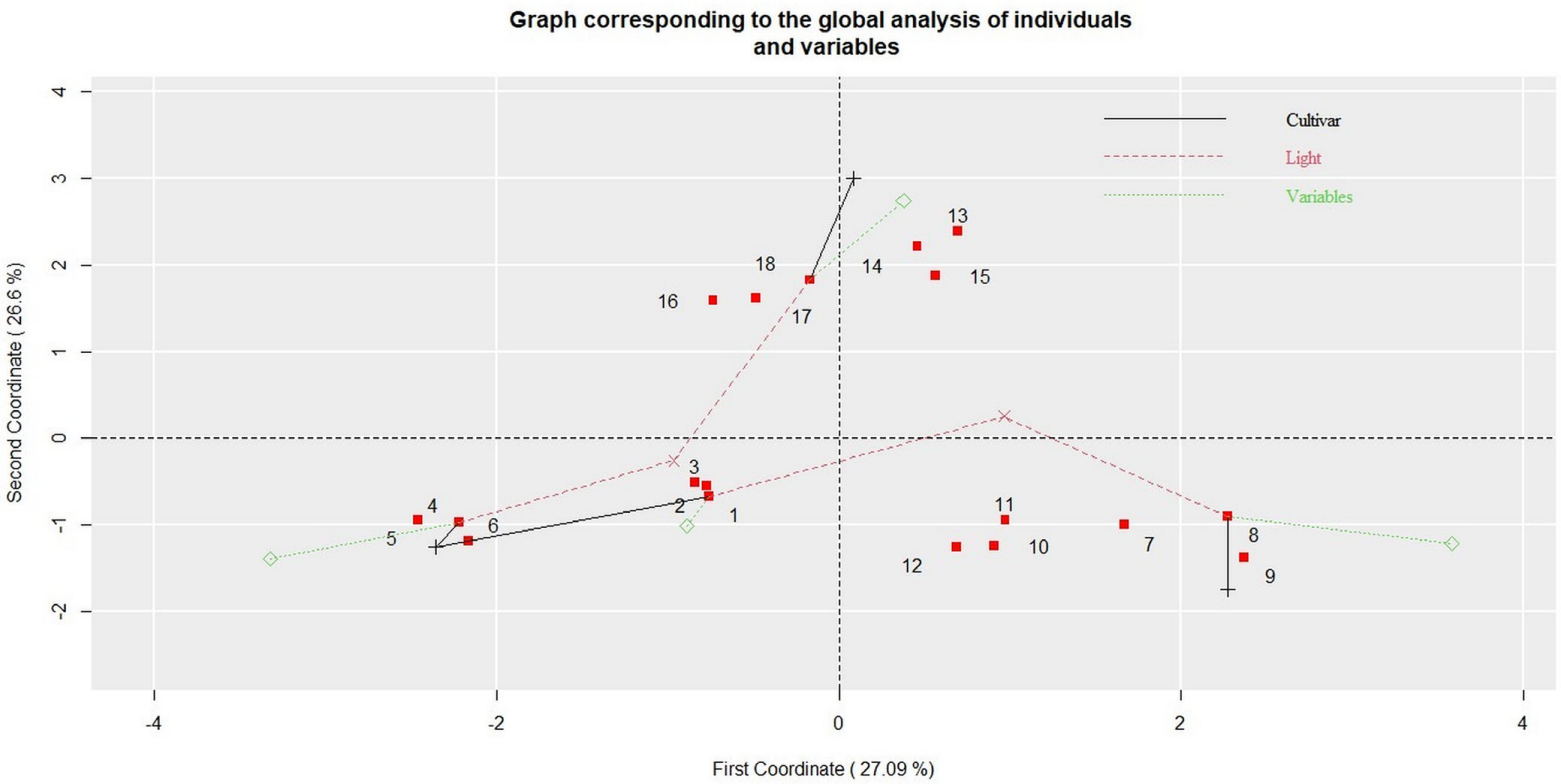
Graph corresponding to the global analysis of individuals and variables.

The results indicate that LS influenced the distribution of individuals, reflecting changes in fruit quality attributes. Distinct positions in the graph reveal different responses among cultivars, suggesting that LS affects not only plant growth but also physicochemical and nutritional characteristics of the fruits.

Global analysis demonstrates that LS and cultivar had distinct effects on fruit quality. The clustering patterns observed reinforce the importance of considering the interaction between these factors to optimize pitaya fruit quality under controlled cultivation conditions.

Significant differences were observed (Table 2) between all pitayas in relation to the evaluation of the variables pH, soluble solids, mass, color (C* and *h*°), firmness, and through the contents of fructose, TFC, TPC, antioxidant activity by ABTS and phosphomolybdenum complex and vitamin C.

**Table 2:** Characteristics of pataya fruits of *Selenicereus* cultivars ‘Boreal Red’, ‘Chinesa’ and ‘Roxa do Pará’

Parameter	‘Boreal Red’	‘Chinesa’	‘Roxa do Pará’	CV%
pH	3.05 ± 0.04b	3.26 ± 0.18bab	3.39 ± 0.27a	6.25
Soluble solids (%)	16.43 ± 0.18a	14.97 ± 0.79b	16.71 ± 0.56a	3.48
Mass (g)	519.84 ± 00.1a	481.69 ± 00.5b	340.69 ± 00.5bc	5.93
Pulp C*	19.08 ± 8.69b	34.08 ± 6.43a	17.96 ± 1.62a	13.70
Peel *h*°	18.07 ± 7.05b	27.72 ± 5.56a	15.42 ± 2.67b	15.62
Peel firmness (N)	35.28 ± 3.58b	53.51 ± 4.78a	54.84 ± 1.23a	7.93
Pulp firmness (N)	3.25 ± 0.16b	3.48 ± 0.22a	3.16 ± 0.17b	6.10
Fructose (g kg^−1^ FW)	49.0 ± 1.28a	38.8 ± 0.54b	35.9 ± 0.41b	14.66
TFC (mg QE kg^−1^ FW)	2981.0 ± 2.07a	2026.0 ± 2.37c	2536.0 ± 5.96b	16.13
TPC (mg GAE kg^−1^ FW)	2128.2 ± 12.12b	2102.9 ± 00.0b	2214.8 ± 9.00a	8.72
ABTS (% of reduction)	46.01 ± 2.13a	40.51 ± 3.36b	48.90 ± 5.92a	9.48
PC (mg AA kg^−1^ FW)	8411.6 ± 00.0b	9416.7 ± 00.0a	5551.5 ± 00.0c	10.32
Vitamin C (mg AA kg^−1^ FW)	405.6 ± 2.57b	467.5 ± 2.81a	230.5 ± 1.65c	6.07

Values are presented as mean ± standard deviation. The mean represents the average of the replicates, while the standard deviation indicates the dispersion of the data around the mean. Means followed by the same letter in the same row do not differ significantly (*P* > 0.05, Tukey's test).

C*, chroma; *h*°, hue angle; TFC, total flavonoid content; TPC, total phenolic content; QE, quercetin equivalents; GAE, gallic acid equivalents; ABTS, 2,2′‐azino‐bis(3‐ethylbenzothiazoline‐6‐sulfonic acid); PC, phosphomolybdenum complex.

According to Table 2, the ‘Boreal Red’ cultivar is different from ‘Roxa do Pará’, and ‘Chinesa’ is similar to the others in relation to pH (*P* < 0.05). The average pH values of the pitayas varied between 3.05 and 3.39, being lower than those found for the red pitaya (*S. polyrhizus*) up to 40 days after anthesis, which varied between 3.63 and 4.48 as reported by Magalhães *et al*.^36^ The variations in pH values may be explained by the reduction of organic acids (citric and malic), which naturally increases pH as the fruit ripens. Additionally, genetic factors influence the accumulation and degradation of these acids, along with environmental and management conditions.^27^, ^37^, ^43^ The higher pH values observed in this study suggest that the pitayas analyzed reached a more advanced and desirable ripening stage compared to those previously reported in the literature, which may indicate favorable cultivation and post‐harvest conditions.

Significant differences (*P* < 0.05) were observed in the soluble solids content of the cultivars, which indicates a relevant variation in the composition of the pitayas. The cultivars ‘Boreal Red’ and ‘Roxa do Pará’ did not differ from each other, with contents of 16.71 and 16.43%, but were statistically different from the ‘Chinesa’ pitaya with a lower content of 14.97%. Factors such as variety, physiological maturity, and cultivation can influence the concentration of the variable even when they are of the same genera, as in the case of *S. undatus*. LS increased the soluble solids contents in all cultivars analyzed. Chen *et al*.^19^ evaluated the flowering response of a red pitaya germplasm collection to LS and observed that the fruits produced in the off‐season reached higher soluble solids contents compared to the fruits produced during the harvest season (*P* < 0.05). These results may indicate that LS contributes to an increase in the concentration of sugars in fruits.

Among the cultivars, ‘Boreal Red’ had the lowest peel firmness with an average value of 35.28 N, compared to ‘Chinesa’ and ‘Roxa do Pará’, which were statistically equal, with values of 53.51 and 54.84, respectively. Regarding the pulp, it was observed that ‘Roxa do Pará’ is softer (3.16 N) than the cultivars ‘Boreal Red’ (3.25 N) and ‘Chinesa’ (3.48 N), being the firmest among the pitaya fruits analyzed (*P* < 0.05). Factors such as the distinct genetic profile that determines the cellular composition of fruits and the ripening stage can impact the observed differences,^36^, ^45^ in addition to the amount of water retained in the cells that influences the turgidity and, consequently, the firmness of the pitaya fruits. Less firm fruits can result in greater susceptibility to physical damage, compromising the structural integrity of the fruits and reducing their shelf life.^54^

The antioxidant capacity reported in pitaya fruit is intrinsically associated with its rich phytochemical composition, such as phenolic compounds, flavonoids, and betacyanins, which have important functions in promoting health.^1^, ^6^, ^24^, ^27^, ^55^, ^56^ Polyphenols are phytochemicals with powerful antioxidant, anti‐inflammatory, anticancer, antimicrobial, antineurodegenerative, and hematoprotective properties, and act in the prevention of obesity, diabetes, cardiovascular diseases, and infections.^57^, ^58^ In fruits, polyphenols, in addition to their protective action, contribute significantly to the formation of flavor and color.^59^

Regarding the comparison of TPC among cultivars (Table 2), statistically significant differences were observed (*P* < 0.05). The cultivar ‘Roxa do Pará’ (2214.8 mg GAE kg^−1^ FW) showed the highest content, followed by ‘Boreal Red’ (2128.2 mg GAE kg^−1^ FW) and ‘Chinesa’ (2102.9 mg GAE kg^−1^ FW), which did not differ statistically from each other. Other studies on pitaya fruits report TPC values ranging from 229.0 mg GAE kg^−1^ (S. *megalanthus*) to 1330.6 mg GAE kg^−1^ (*S*. *polyrhizus*, cv. ‘Royal Red’).^52^, ^60^ Therefore, the three cultivars evaluated in this study can be considered rich sources of these bioactive compounds.

Many groups make up the polyphenol class, and among these are flavonoids. These secondary metabolites with strong antioxidant activity play essential roles in mediating plant responses to environmental factors. In fruits, they also participate in different biological processes, such as coloration.^61^, ^62^ TFC was not influenced by LS in the evaluated cultivars; however, statistically significant differences were observed among cultivars (*P* < 0.05). The cultivar ‘Boreal Red’ showed the highest content (298.1 mg QE kg^−1^ FW), followed by ‘Roxa do Pará’ and ‘Chinesa’, with 253.6 and 202.6 mg QE kg^−1^ FW, respectively. Other studies have reported total flavonoid contents ranging from 48.4 to 193.6 mg QE kg^−1^ FW in *S. undatus*.^63^, ^64^ Therefore, all evaluated cultivars can be considered rich in total flavonoids; however, LS was not effective for this parameter. Supplemental blue light has also been reported to increase flavonoid accumulation in pitaya fruits, indicating that the response of these compounds may depend on the light spectrum, duration of exposure, and genotype.^40^ The antioxidant activity of pitaya results from the synergistic action of several compounds, including phenolics, flavonoids, betalains, and ascorbic acid. Therefore, differences in phenolic and flavonoid contents do not necessarily lead to proportional changes in overall antioxidant capacity.

No significant differences (*P* > 0.05) were observed in the antioxidant power among the cultivars ‘Boreal Red’, ‘Roxa do Pará’, and ‘Chinesa’ in LS and ALS treatments, as determined by the ABTS and phosphomolybdenum complex methods. Although numerical variations were detected, they were not statistically relevant. Enhanced antioxidant responses under supplemental blue light have also been reported in pitaya, including increased radical‐scavenging capacity and antioxidant enzyme activity.^40^ The ‘Roxa do Pará’ pitaya, followed by ‘Borel Red’, stood out in the ALS treatment in relation to the two antioxidant analyses performed, with emphasis on the ‘Roxa do Pará’ pitaya, which showed a reduction power of 48.97% in the ABTS method and 922.16 mg AA 100 g^−1^ FW in the phosphomolybdenum complex. This last method is based on the evaluation of the total antioxidant activity through the reduction of Mo(VI) to Mo(V), in the presence of electro donor compounds in the sample, and subsequent formation of the phosphomolybdenum complex, at acidic pH and considerably high temperature.^30^ In an experiment with pitaya fruit juices (*S. polyrhizus*) at different concentrations, Manan *et al*.^65^ identified a reduction capacity by the ABTS method ranging from 38.69% to 92.66%, and a phosphomolybdenum complex content ranging from 7.81 to 28.94 mg AA 100 mL^−1^ of juice.

Regarding vitamin C content, the cultivar ‘Chinesa’ presented the highest value (467.5 mg AA kg^−1^ FW), followed by ‘Boreal Red’ and ‘Roxa do Pará’, with 405.6 and 230.5 mg AA kg^−1^ FW, respectively. Studies on different pitaya fruit species have reported vitamin C contents ranging from 101.3 mg AA kg^−1^ in *S. ocamponis* to 200.0 mg AA kg^−1^ in *S. polyrhizus* and 311.1 mg AA kg^−1^ in *S. undatus*.^66^, ^67^ The ‘Chinesa’ cultivar also showed the highest ascorbic acid content under ALS conditions (470.5 mg AA kg^−1^ FW), with values comparable to those found in orange, a major dietary source of vitamin C for humans. The recommended daily amount varies depending on factors such as age, sex, and health conditions of the individual.^68^ For adults, the recommendations are 75 mg for women and 90 mg for men.^68^, ^69^ The cultivars ‘Boreal Red’ and ‘Roxa do Pará’ presented a lower ascorbate content than the ‘Chinesa’ variety. However, pitaya fruits with LS showed a significant increase in ascorbic acid compared to fruits without supplementation. Similar improvements in nutritional quality under supplemental light have been reported in pitaya fruits.^40^

For future studies, approaches such as investigation with different wavelengths and the effects of LS over several harvests and how this use affects the plant's lifespan may be of great interest to the scientific community and farmers related to fruit growing. Recent metabolomic studies have also shown that supplemental light alters volatile compounds associated with fruit flavor, indicating potential effects on sensory quality beyond the physicochemical parameters evaluated in the present study.^40^ In addition to the economic viability, the cost–benefit of implementing large‐scale artificial lighting systems should also consider energy efficiency, installation and maintenance costs, and the associated environmental impact.

## CONCLUSIONS

The findings of this study demonstrate that LS during flowering significantly influences fruit quality in pitaya. Increases in fruit mass, sugar content, and vitamin C highlight the potential of this technique to enhance the nutritional and commercial value of the fruit. Among the evaluated cultivars, ‘Boreal Red’ showed the most consistent positive response, particularly in terms of fruit mass and sugar accumulation, indicating its greater potential for cultivation under supplemental lighting conditions. The cultivars ‘Chinesa’ and ‘Roxa do Pará’ also responded to LS, although the effects varied depending on the evaluated parameter. Under short photoperiod conditions, LS represents a promising strategy to improve fruit quality and enable off‐season production. Further studies are needed to evaluate its effects on yield under these conditions.

## CONFLICT OF INTEREST

The authors declare that they have no known competing financial interests or personal relationships that could appear to influence the work reported in this paper.

## ETHICS STATEMENT

The authors declare that all experiments in this study were conducted according to the established ethical guidelines.

## AUTHOR CONTRIBUTIONS

Luíz Guilherme Malaquias da Silva: conceptualization; methodology; investigation; software; data curation; writing – original draft. Carlos Alexandre Rocha da Costa: methodology; investigation; writing – review and editing. Giovanni Aleixo Batista: methodology. Estela Corrêa de Azevedo: methodology. Fabrício Teixeira de Lima Gomes: methodology, investigation. Laryssa Ribeiro Calcagnoto: methodology; investigation; data curation. Gabriel Luis Castiglioni: methodology; investigation. Marcelo Ângelo Cirillo: methodology; investigation; data curation. Danilo José Machado de Abreu: resources; writing – review and editing; supervision. Flávia Matias Oliveira da Silva: resources; writing – review and editing; supervision. Katiuscia Alves Amorim: data curation; writing – review and editing. Leila Aparecida Salles Pio: resources; writing – review and editing; supervision. Eduardo Valério de Barros Vilas Boas: writing – review and editing; supervision. Elisangela Elena Nunes Carvalho: writing – review and editing; supervision.

## Data Availability

The data that support the findings of this study are available on request from the corresponding author. The data are not publicly available due to privacy or ethical restrictions.
